# Exploration of Cognitive Outcomes and Risk Factors for Cognitive Decline Shared by Couples

**DOI:** 10.1001/jamanetworkopen.2021.39765

**Published:** 2021-12-20

**Authors:** Hee Won Yang, Jong Bin Bae, Dae Jong Oh, Dong Gyu Moon, Eunji Lim, Jin Shin, Bong Jo Kim, Dong Woo Lee, Jeong Lan Kim, Jin Hyeong Jhoo, Joon Hyuk Park, Jung Jae Lee, Kyung Phil Kwak, Seok Bum Lee, Seok Woo Moon, Seung-Ho Ryu, Shin Gyeom Kim, Ji Won Han, Ki Woong Kim

**Affiliations:** 1 Department of Neuropsychiatry, Seoul National University Bundang Hospital, Seongnam, South Korea; 2 Department of Psychiatry, Seoul National University, College of Medicine, Seoul, South Korea; 3 Department of Psychiatry, SMG-SNU Boramae Medical Center, Seoul, South Korea; 4 Department of Psychiatry, Gyeongsang National University Changwon Hospital, Changwon, South Korea; 5 Department of Psychiatry, Gyeongsang National University, School of Medicine, Jinju, South Korea; 6 Department of Neuropsychiatry, Inje University Sanggye Paik Hospital, Seoul, South Korea; 7 Department of Psychiatry, School of Medicine, Chungnam National University, Daejeon, South Korea; 8 Department of Neuropsychiatry, Kangwon National University Hospital, Chuncheon, South Korea; 9 Department of Neuropsychiatry, Jeju National University Hospital, Jeju, South Korea; 10 Department of Psychiatry, Dankook University Hospital, Cheonan, South Korea; 11 Department of Psychiatry, Dongguk University Gyeongju Hospital, Gyeongju, South Korea; 12 Department of Psychiatry, School of Medicine, Konkuk University, Konkuk University Chungju Hospital, Chungju, South Korea; 13 Department of Psychiatry, School of Medicine, Konkuk University, Konkuk University Medical Center, Seoul, South Korea; 14 Department of Neuropsychiatry, Soonchunhyang University Bucheon Hospital, Bucheon, South Korea; 15 Department of Brain and Cognitive Science, Seoul National University College of Natural Sciences, Seoul, South Korea

## Abstract

**Importance:**

Although couples could share many risk factors of cognitive disorders in their lifetime, whether shared risk factors mediate the shared risk of cognitive disorders has rarely been investigated.

**Objective:**

To identify the risk factors of cognitive decline shared within couples and investigate their mediating roles in the shared risk of cognitive disorders and cognitive functions within couples.

**Design, Setting, and Participants:**

A prospective cohort study was launched in November 1, 2010, and 784 participants were followed up every 2 years until December 31, 2020. This nationwide, multicenter, community-based study included older couples from the Korean Longitudinal Study on Cognitive Aging and Dementia (KLOSCAD) and a cohort of their spouses (KLOSCAD-S).

**Exposures:**

The cognitive disorder of a spouse was defined as mild cognitive impairment or dementia.

**Main Outcomes and Measures:**

The mediating roles of factors shared within couples on the association between one spouse’s cognitive disorder and the other’s risk of cognitive disorders was examined with structural equation modeling.

**Results:**

Included were 784 KLOSCAD participants (307 women [39.2%] and 477 men [60.8%]; mean [SD] age, 74.8 [4.8] years) and their spouses (477 women [60.8%] and 307 men [39.2%]; mean [SD] age, 73.6 [6.2] years). The cognitive disorder of the KLOSCAD participants was associated with almost double the risk of cognitive disorder of their spouses in the KLOSCAD-S cohort (odds ratio, 1.74; 95% CI, 1.12-2.69; *P* = .01). History of head injury (β = 0.50; 95% CI, 0.09-0.90; *P* = .02) and age (β = 2.57; 95% CI, 1.37-3.76; *P* < .001) mediated the association between cognitive disorder in the KLOSCAD participants and their spouses’ risk of cognitive disorder. Physical inactivity mediated the association through major depressive disorder (β = 0.33, 95% CI, 0.09-0.57, *P* = .006 for physical inactivity; β = 0.28, 95% CI, 0.13-0.44, *P* < .001 for major depressive disorder). These factors similarly mediated the association between spousal cognitive disorder and cognitive functions such as memory and executive function.

**Conclusions and Relevance:**

These findings suggest that the risk factors shared within couples may mediate approximately three-quarters of the spousal risk of cognitive disorders. Identification of and intervention in the shared risk factors of dementia within couples may reduce the risk of cognitive disorders in the spouses of people with dementia.

## Key Points

**Question:**

Do the risk factors shared within couples mediate their shared risk of cognitive disorders?

**Finding:**

In this cohort study of 784 older couples, physical inactivity, major depressive disorder, and a history of head injury that were shared within couples mediated almost half of the spousal risk of cognitive disorder.

**Meaning:**

The findings of this study suggest that identification of and intervention in the shared risk factors of dementia within couples may reduce the risk of cognitive disorders in the spouses of people with dementia.

## Teaser

This cohort study uses Korean Longitudinal Study on Cognitive Aging and Dementia data to identify and investigate shared risk factors of dementia within older couples.

## Introduction

Spouses of individuals with dementia are known to be at higher risk of dementia and cognitive decline in global cognition, executive function, memory, and language.^1,2,3,4,5,6,7,8,9,10,11,12^ Several mediators, including depression, sleep problems, social isolation, exercise, diet, metabolic syndrome, and inflammation, may predispose spousal caregivers of patients with dementia to cognitive decline and possibly dementia.^13^ In addition, there is evidence for similarities in cognitive function within couples.^14,15,16^ Although higher rates of glucose, insulin resistance, and obesity have been suggested as possible mediators, other potential factors that can explain these similarities need to be investigated.^12^

Spouses generally share a common environment, and many studies have investigated spousal concordances for factors such as lifestyle and physical and psychological health.^17,18,19,20,21,22,23^ These factors, which are concordant within couples, are known to be associated with a risk of dementia or cognitive decline^24^ and are also negatively associated with spousal cognitive disorders.^13,25,26,27,28,29^ Therefore, these factors may mediate cognitive disorders and changes in cognitive functions that correlate within couples. In addition, because most of these factors are modifiable and can contribute to a reduction in the risk of dementia,^24^ early detection and correction within couples is important in preventing dementia. However, to our knowledge, the mediating role of shared risk factors within couples on the risk of cognitive impairment associated with spousal cognitive disorders has never been directly investigated.

Our objective was to identify the risk factors shared within couples and examine their mediating roles in the shared risk of cognitive disorders and cognitive functions within couples in a population-based, couple cohort study.

## Methods

### Study Design and Participants

We acquired data for this study from the Korean Longitudinal Study on Cognitive Aging and Dementia (KLOSCAD).^30^ The KLOSCAD is an ongoing, nationwide, multicenter, prospective cohort study on 6818 community-dwelling Koreans aged 60 years or older who were randomly sampled from the residents of 13 districts across South Korea with the national residential roster at the end of 2009. The baseline assessment was conducted from November 1, 2010, to October 31, 2012, and follow-up assessments were conducted every 2 years until December 31, 2020. At the fourth follow-up assessment (from January 1, 2019, to December 31, 2020), we constructed a spousal cohort (KLOSCAD-S) consisting of the spouses of the KLOSCAD participants.

The protocol for this study was explained to all participants, and each participant provided written informed consent. The study protocol was approved by the institutional review board of the Seoul National University Bundang Hospital. This report followed the Strengthening the Reporting of Observational Studies in Epidemiology (STROBE) reporting guideline for cohort studies.

### Assessment of Covariates

Research nurses evaluated the participants’ demographic characteristics (age, sex, and educational level), physical comorbidities (diabetes, hypertension, hearing loss, and head trauma), alcohol consumption, smoking, and physical activity. History of heavy alcohol use was defined as the average lifetime amount of alcohol use over 21 standard units per week (1 standard unit is approximately 10 g of pure alcohol). We defined a history of exposure to smoking as the cumulative amount of one’s own smoking or spousal smoking that was greater than 10 pack-years during a concurrent relationship. Physical inactivity was defined as less than 2.5 hours of moderate activity per week and less than 1.25 hours of vigorous activity per week, according to the World Health Organization’s recommendations on the minimum amount of activity that confers health benefits.^31^ We evaluated the burden of comorbid chronic medical illnesses with the Cumulative Illness Rating Scale.^32^ The scale combines the morbidity of chronic medical problems of 14 organ systems, and the association among chronic conditions of each system is rated from 0 to 4, with the sum of all ratings amounting to the cumulative scale score. This scale is among the most valid and reliable measures of multimorbidity and has been reported to be a valid indicator of health status in geriatric patients.^33^ Height and body weight were measured by trained research nurses. Each participant’s body mass index was calculated as weight in kilograms divided by height in meters squared,^2^ with greater than or equal to 25.0 defining obesity in accordance with World Health Organization guidelines for the Asia-Pacific region.^34^

Geriatric psychiatrists assessed all participants through face-to-face standardized diagnostic interviews using the Korean version of the Mini-International Neuropsychiatric Interview.^35^ They obtained any history of mood disorders from the participants or their family members. A panel of geriatric psychiatrists diagnosed current major depressive disorder (MDD) and minor depressive disorder with the *Diagnostic and Statistical Manual of Mental Disorders, Fourth Edition (DSM-IV)* criteria.^36^ We also evaluated the severity of depressive symptoms with the revised Korean version of the Geriatric Depression Scale.^37^

### Cognitive Assessments

Geriatric psychiatrists conducted face-to-face standardized diagnostic interviews and performed physical and neurologic examinations of every participant by using the Korean version of the Consortium to Establish a Registry for Alzheimer Disease Assessment Packet Clinical Assessment Battery (CERAD-K-C).^38^ Trained research neuropsychologists administered neuropsychological tests that consisted of the CERAD-K Neuropsychological Assessment Battery (CERAD-NP)^39^ and the Korean version of the Frontal Assessment Battery.^40^ From the CERAD-NP, we calculated the CERAD memory score (CERAD-MS) by summing the word list recall test score (maximum, 10), word list recognition test score (maximum, 10), and the average score of 3 trials in the word list memory test (maximum, 10) for each participant. We defined cognitive disorders as mild cognitive impairment or dementia. Through diagnostic consensus conferences, a panel of geriatric psychiatrists diagnosed dementia and mild cognitive impairment according to *DSM-IV* criteria^36^ and the consensus criteria proposed by the International Working Group on Mild Cognitive Impairment,^41^ respectively.

### Statistical Analysis

We compared the demographic and clinical characteristics between couples in which the KLOSCAD participants had cognitive disorders and couples in which the KLOSCAD participants did not have a cognitive disorder, using Pearson χ^2^ tests for categorical variables and *t* tests for continuous variables. We examined the agreement of the demographic and clinical characteristics within couples by using the intraclass correlation coefficient for continuous variables and the κ coefficient for categorical variables. To examine the association between spousal cognitive disorders and the risk of cognitive disorders, we used binary logistic regression analyses. To examine the association of spousal cognitive disorders with memory and executive function, we conducted analysis of covariance. We then examined the mediating role of the factors shared within couples on the association between spousal cognitive disorders and the risk of cognitive disorders and cognitive functions, using structural equation modeling. The structural equation model was designed as follows:

*Y* = *c* + ε*X* + β_1_*M*_1_ + β_2_*M*_2_ + β_3_*M*_3_ + ∙∙∙ + *β_k_M_k_*

*M_k_* = *d* + *α_k_X* for *k* = 1, …, *K*

We denoted *X*, *M*, and *Y* as the exposure, mediators, and outcomes, respectively. We included mediator-mediator interactions in the model if the mediators affected one another; for 2 mediators with interaction, the model became:

*M_i_* = *d* + *α_i_X*

*M_j_* = *d* + *α_j_X* + *γijMi*

Then, we estimated the direct and indirect associations between exposure *X* and outcome *Y* as follows:

Direct association = ε

Indirect association = α_1_β_1_ + α_2_β_2_ + α_3_β_3_ + ∙∙∙ *α_i_γijβ_j_* + *α_k_β_k_*

We denoted *c* and *d* as intercepts for each equation; *α* is a coefficient; *k* indicates the order of mediators, of which *i* is an example as a mediator that has no interactions with other variables; and *γij *is a coefficient representing the interaction between the *i*th mediator and the *j*th mediator.

We performed all statistical analyses with R version 4.0.3 (R Core Team). *P* values were 2-sided, with *P* < .05 considered statistically significant.

## Results

We included 784 KLOSCAD participants (307 women [39.2%] and 477 men [60.8%]; mean [SD] age, 74.8 [4.8] years) and their spouses (477 women [60.8%] and 307 men [39.2%]; mean [SD] age, 73.6 [6.2] years) in the current study (Table 1). All couples were heterosexual, and the KLOSCAD-S participants were younger but less educated than the KLOSCAD participants. Among the 784 KLOSCAD participants, 121 (15.4%) had cognitive disorders. The participants in the KLOSCAD-S group whose spouses in the KLOSCAD had cognitive disorders (SCD+) were older, less educated, less physically active, and more likely to have a history of head injury compared with the KLOSCAD-S participants whose spouses in the KLOSCAD did not have a cognitive disorder (SCD−).

**Table 1.** Demographic and Clinical Characteristics of the Participants

Characteristic	KLOSCAD			KLOSCAD-S		
	No. (%)		*P* value^a^	No. (%)		*P* value^a^
	CD+ (n = 121)	CD− (n = 663)		SCD+ (n = 121)	SCD− (n = 663)	
Age, mean (SD), y	77.3 (5.4)	74.4 (4.6)	<.001	75.7 (6.0)	73.2 (6.1)	<.001
Education, mean (SD), y	9.1 (5.1)	10.5 (5.0)	.005	8.7 (4.8)	10.1 (5.0)	.006
Women	47 (38.8)	260 (39.2)	.94	74 (61.2)	403 (60.8)	>.99
Men	74 (61.2)	403 (60.8)	>.99	47 (38.8)	260 (39.2)	.94
Hearing loss	17 (14.0)	24 (3.6)	<.001	8 (6.6)	35 (5.3)	.55
History of head injury	9 (7.4)	41 (6.2)	.60	8 (6.6)	15 (2.3)	.02
BMI, mean (SD)	23.9 (3.3)	24.7 (3.1)	.01	24.1 (3.8)	24.3 (3.2)	.67
Obesity^b^	51 (42.1)	290 (43.7)	.75	49 (40.5)	258 (38.9)	.74
Lifetime amount of alcohol use, mean (SD), SU/wk	3.3 (8.7)	2.6 (7.6)	.33	3.6 (10.5)	2.6 (9.7)	.30
History of heavy alcohol use^c^	14 (11.6)	85 (12.8)	.70	11 (9.1)	59 (8.9)	.95
Lifetime amount of smoking, mean (SD), pack-years	16.8 (25.7)	15.1 (23.1)	.46	9.8 (20.7)	9.4 (20.9)	.83
History of exposure to smoking^d^	80 (66.1)	397 (59.9)	.20	80 (66.1)	393 (59.3)	.16
Metabolic equivalent task, mean (SD), h/wk^e^	15.6 (29.0)	26.0 (36.1)	.003	16.4 (23.5)	22.5 (31.2)	.04
Physical inactivity^f^	69 (57.0)	186 (28.1)	<.001	60 (49.6)	223 (33.6)	.001
Hypertension	77 (63.6)	388 (58.5)	.29	59 (48.8)	350 (52.8)	.41
Diabetes	35 (28.9)	149 (22.5)	.12	25 (20.7)	142 (21.4)	.85
Cumulative Illness Rating Scale, mean (SD), points^g^	1.7 (0.3)	1.6 (0.4)	.04	1.6 (0.4)	1.5 (0.4)	.18
Geriatric Depression Scale, mean (SD), points^h^	11.2 (7.2)	7.5 (5.9)	<.001	9.7 (6.5)	8.2 (6.2)	.01
Major depressive disorder^i^	17 (14.0)	46 (6.9)	.008	11 (9.1)	42 (6.3)	.27
CERAD-MS, mean (SD), points^j^	14.0 (6.1)	21.3 (3.8)	<.001	16.5 (5.6)	18.6 (4.7)	<.001
Frontal Assessment Battery, mean (SD), points^k^	11.5 (4.2)	15.3 (2.5)	<.001	12.8 (4.1)	14.2 (3.1)	.001
Cognitive disorder^l^	121 (100.0)	0	<.001	47 (38.8)	150 (22.6)	<.001

Abbreviations: BMI, body mass index (calculated as weight in kilograms divided by height in meters squared); CD−, KLOSCAD participants without cognitive disorders; CD+, KLOSCAD participants with cognitive disorders; CERAD-MS, Consortium to Establish a Registry for Alzheimer Disease memory score, calculated by summing the participants’ word list recall test scores (maximum score, 10), the word list recognition test scores (maximum score, 10), and the average of 3 trials in the word list memory test (maximum score, 10); KLOSCAD-S, Korean Longitudinal Study on Cognitive Aging and Dementia spouse cohort; SCD−, spouses of CD− participants; SCD+, spouses of CD+ participants; SU, standard unit (1 SU is approximately 10 g of pure alcohol).

^a^ *t* Test for continuous variables and χ^2^ test for categorical variables.

^b^ Body mass index greater than or equal to 25.

^c^ Average lifetime amount of alcohol use at least 21 SU/wk.

^d^ Cumulative amount of smoking or spousal smoking during current marriage greater than 10 pack-years.

^e^ The ratio of metabolic rate during a specific physical activity to a reference metabolic rate, set by convention to 3.5 mL of oxygen·per kilogram per·minute. Total metabolic equivalent task hours per week equals the sum of light (3.0 × light-intensity activity hours × walking days) + moderate (4.5 × moderate-intensity activity hours × moderate days) + vigorous (8.0 × vigorous-intensity activity hours × vigorous-intensity days) metabolic equivalent task hours per week.

^f^ Less than 2.5 hours of moderate activity per week and less than 1.25 hours of vigorous activity per week.

^g^ The Cumulative Illness Rating Scale ranges from 0 to 4 points, with higher scores indicating higher disease burden or severity.

^h^ The Geriatric Depression Scale ranges from 0 to 30 points, with higher scores indicating higher depression severity.

^i^ Current or a history of major depressive disorder according to *Diagnostic and Statistical Manual of Mental Disorders, Fourth Edition* criteria.

^j^ Sum of each participant’s word list recall test scores (maximum score, 10), word list recognition test score (maximum score, 10), and the average of 3 trials in the word list memory test (maximum score, 10).

^k^ Frontal Assessment Battery scores range from 0 to 18 points, whith higher scores indicating better performance.

^l^ Mild cognitive impairment or dementia.

Both the SCD+ and SCD− groups showed high concordance with their spouses in terms of age, educational level, smoking, physical inactivity, and Geriatric Depression Scale score. In addition, the SCD− group showed high concordance with their spouses in terms of their history of head injury and MDD (Table 2).

**Table 2.** Agreement of Demographic and Clinical Characteristics Within Couples

Characteristic	CD+ and SCD+ (n = 121)			CD− and SCD− (n = 663)		
	No. (%)		*P* value	No. (%)		*P* value
	Absolute differences	Concordance^a^		Absolute differences	Concordance^a^	
Age, mean (SD), y	1.5 (4.9)	0.617	<.001	1.2 (4.7)	0.611	<.001
Education, mean (SD), y	0.3 (4.4)	0.616	<.001	0.4 (4.7)	0.557	<.001
Hearing loss	9 (7.4)	−0.011	.90	−11 (−1.7)	−0.009	.80
History of head injury	1 (0.8)	−0.075	.41	26 (3.9)	0.150	<.001
BMI, mean (SD)	−0.5 (4.5)	0.041	.33	0.4 (4.2)	0.058	.07
Obesity^b^	2 (1.7)	0.046	.61	32 (4.8)	0.063	.10
Lifetime amount of alcohol use, mean (SD), SU/wk	−0.3 (14.1)	−0.076	.79	0.0 (12.4)	−0.005	.56
History of heavy alcohol use^c^	3 (2.5)	−0.113	.21	26 (3.9)	−0.071	.06
Lifetime amount of smoking, mean (SD), pack-years	6.9 (37.6)	−0.292	>.99	5.7 (35.0)	−0.253	>.99
History of exposure to smoking^d^	0	1.000	<.001	4 (0.6)	0.969	<.001
Metabolic equivalent task, mean (SD), h/wk	−0.9 (36.5)	0.045	.31	3.6 (41.5)	0.2	<.001
Physical inactivity^e^	9 (7.4)	0.191	.03	−37 (−5.6)	0.137	<.001
Hypertension	18 (14.9)	−0.018	.84	38 (5.7)	−0.023	.55
Diabetes	10 (8.3)	0.034	.70	7 (1.1)	0.054	.17
Cumulative Illness Rating Scale, mean (SD), points^f^	0.1 (0.5)	0.095	.15	0.1 (0.02)	0.043	.13
Geriatric Depression Scale, mean (SD), points^g^	1.6 (8.2)	0.262	.002	−0.8 (7.4)	0.242	<.001
Major depressive disorder^h^	6 (5.0)	0.036	.68	4 (0.6)	0.099	.01

Abbreviations: BMI, body mass index (calculated as weight in kilograms divided by height in meters squared); CD−, Korean Longitudinal Study on Cognitive Aging and Dementia (KLOSCAD) participants without cognitive disorders; CD+, KLOSCAD participants with cognitive disorders; SCD−, spouses of CD− participants; SCD+, spouses of CD+ participants; SU, standard unit (1 SU is approximately 10 g of pure alcohol).

^a^ Intraclass correlation coefficient for continuous variables and κ coefficients for categorical variables.

^b^ Body mass index (calculated as weight in kilograms divided by height in meters squared) greater than or equal to 25.

^c^ Average lifetime amount of alcohol use at least 21 SU/wk.

^d^ Cumulative amount of smoking or spousal smoking during current marriage greater than 10 pack-years.

^e^ Less than 2.5 hours of moderate activity per week and less than 1.25 hours of vigorous activity per week.

^f^ The Cumulative Illness Rating Scale ranges from 0 to 4 points, with higher scores indicating higher disease burden or severity.

^g^ The Geriatric Depression Scale ranges from 0 to 30 points, with higher scores indicating higher depression severity.

^h^ Current or a history of major depressive disorder according to *Diagnostic and Statistical Manual of Mental Disorders, Fourth Edition* criteria.

Cognitive disorders were more prevalent in the SCD+ group than in the SCD− group, indicating that they were associated with a spouse’s risk of cognitive disorder (47/121 [38.8%] vs 150/663 [22.6%]; *P* < .001) (Table 1). In the logistic regression models, the cognitive disorder of the KLOSCAD participants was associated with almost double the risk of cognitive disorder in their spouses in the KLOSCAD-S cohort (odds ratio, 1.74; 95% CI, 1.12-2.69; *P* = .01) (Table 3). This association remained significant when the factors that were concordant within couples and those that were not were additionally adjusted. None of the factors that were not concordant within couples were associated with the risk of cognitive disorders in the KLOSCAD-S participants. However, among the 4 factors (physical inactivity, exposure to smoking, history of head injury, and MDD) that were concordant within couples, physical inactivity, history of head injury, and MDD were independently associated with the risk of cognitive disorders in the KLOSCAD-S participants.

**Table 3.** Association of Spousal Cognitive Disorders on the Risk of Cognitive Disorders

Variable	Model 1^a^		Model 2^a^			Model 3^a^	
	OR (95% CI)	*P* value	OR (95% CI)	*P* value	OR (95% CI)		*P* value
Cognitive disorder of spouse^b^	1.93 (1.27-2.92)	.002	1.97 (1.29-2.99)	.002	1.74 (1.12-2.69)		.01
Unshared factors							
Hearing loss	NA	NA	1.80 (0.93-3.47)	.08	1.80 (0.92-3.53)		.09
Obesity^c^	NA	NA	1.10 (0.78-1.56)	.59	1.10 (0.77-1.57)		.61
Heavy alcohol use^d^	NA	NA	1.44 (0.84-2.47)	.19	1.27 (0.72-2.25)		.42
Hypertension	NA	NA	0.94 (0.66-1.33)	.72	0.95 (0.66-1.35)		.77
Diabetes	NA	NA	1.34 (0.90-1.98)	.15	1.31 (0.87-1.98)		.20
Shared factors							
Physical inactivity^e^	NA	NA	NA	NA	1.48 (1.03-2.13)		.03
Exposure to smoking^f^	NA	NA	NA	NA	0.94 (0.67-1.34)		.75
History of head injury	NA	NA	NA	NA	4.60 (1.87-11.31)		.001
Major depressive disorder^g^	NA	NA	NA	NA	3.88 (2.10-7.15)		<.001

Abbreviations: NA, not applicable; OR, odds ratio.

^a^ Binary logistic regression analysis adjusted for age and education.

^b^ Mild cognitive impairment or dementia.

^c^ Body mass index (calculated as weight in kilograms divided by height in meters squared) greater than or equal to 25.

^d^ Average lifetime amount of alcohol use at least 21 standard units (SU)/wk (1 SU is approximately 10 g of pure alcohol).

^e^ Less than 2.5 hours of moderate activity per week and less than 1.25 hours of vigorous activity per week.

^f^ Cumulative amount of one’s own smoking or spousal smoking during current marriage greater than 10 pack-years.

^g^ Current or a history of major depressive disorder according to *Diagnostic and Statistical Manual of Mental Disorders, Fourth Edition* criteria.

In the structural equation model with multiple mediators (Figure, A), history of head injury (standardized coefficient β = 0.50; 95% CI, 0.09-0.90; *P* = .02) and age (β = 2.57; 95% CI, 1.37-3.76; *P* < .001) mediated the association between cognitive disorder in the KLOSCAD participants and their spouses’ risk of cognitive disorder. Physical inactivity mediated the association via MDD (β = 0.33, 95% CI, 0.09-0.57; *P* = .006 for physical inactivity; β = 0.28, 95% CI, 0.13-0.44; *P* < .001 for MDD). Major depressive disorder did not mediate the association (β = 0.13; 95% CI, −0.23 to 0.48; *P* = .49) but was independently associated with increased risk of cognitive disorder (β = 0.35; 95% CI, 0.18-0.53; *P* < .001). Education indirectly mediated the association between spousal cognitive disorders and the risk of cognitive disorders by increasing inactivity and MDD (β = −1.36, 95% CI, −2.34 to −0.38, *P* = .007 for education; β = −0.06, 95% CI, −0.08 to −0.04, *P* < .001 for physical inactivity; β = −0.03, 95% CI, −0.06 to −0.01, *P* = .02 for MDD). Cognitive disorder in the KLOSCAD participants was associated with their spouses’ risk of cognitive disorder indirectly by 7.4% (0.033/0.447) through physical inactivity via MDD and 42.3% (0.189/0.447) through history of head injury. However, there was no association between spousal cognitive disorders and the risk of cognitive disorders (β = 0.11; 95% CI, −0.20 to 0.41; *P* = .49).

**Figure.**
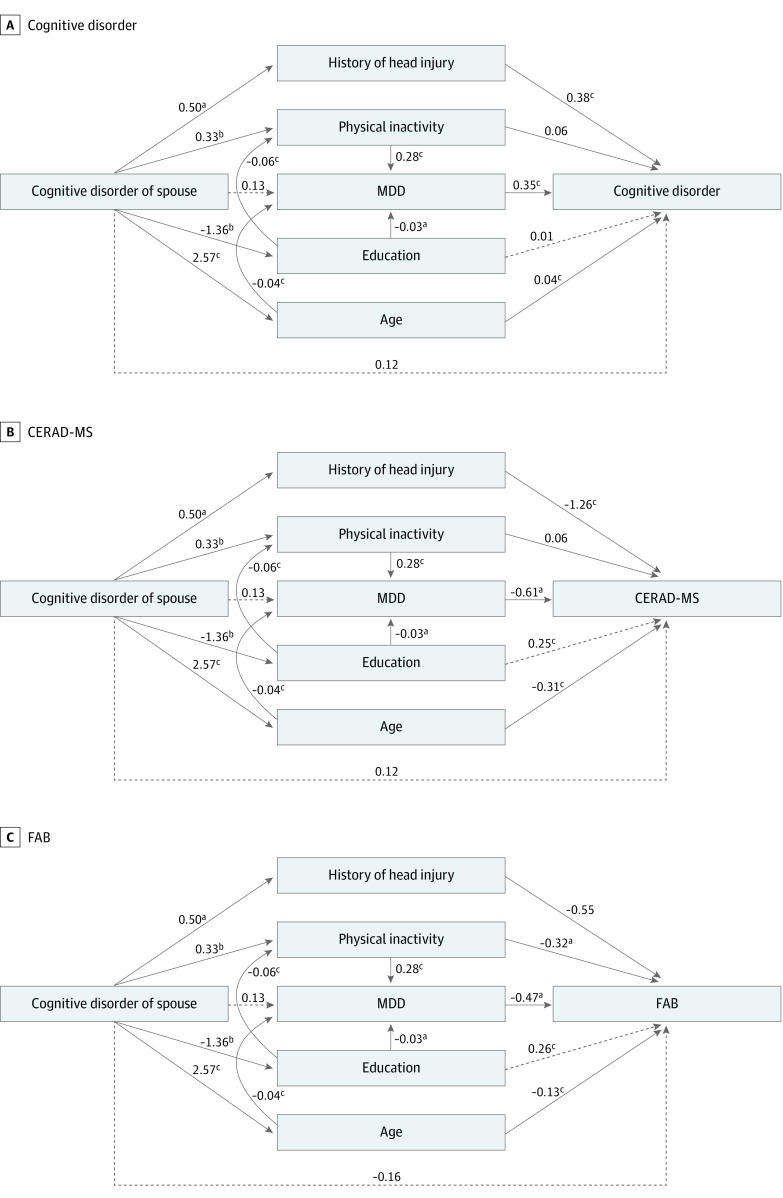
Mediating Role of Factors Shared Within Couples in the Shared Risk of Cognitive Disorders and Cognitive Functions Direct and indirect associations between spousal cognitive disorders and the risk of cognitive disorders are shown, along with standardized coefficients. Dashed lines indicate nonsignificant pathways. CERAD-MS indicates Consortium to Establish a Registry for Alzheimer Disease memory score, calculated by summing the participants’ word list recall test scores (maximum score, 10), the word list recognition test scores (maximum score, 10), and the average of 3 trials in the word list memory test (maximum score, 10); FAB, Frontal Assessment Battery; MDD, major depressive disorder. ^a^*P* < .05. ^b^*P* < .01. ^c^*P* < .001.

The CERAD-MS and Frontal Assessment Battery scores of the SCD+ group were lower than those of the SCD− group (Table 1). They were associated with the cognitive disorder of the spouses in the KLOSCAD cohort, which remained significant when age, educational level, and the factors that were not concordant within couples were adjusted. However, this association was not statistically significant when the factors that were concordant within couples were additionally adjusted (Table 4). Similar to that in the structural equation models presented earlier, physical inactivity mediated the association between the KLOSCAD participants’ cognitive disorders and their spouses’ performance in both the CERAD-MS and Frontal Assessment Battery through the association with MDD. A history of head injury mediated the association between the KLOSCAD participants’ cognitive disorders and their spouses’ performance in the CERAD-MS only. Educational level and age also mediated the association. However, there were no associations between spousal cognitive disorders and memory and executive function.

**Table 4.** Associations Between Spousal Cognitive Disorders and Memory and Executive Function

	Model 1^a^		Model 2^b^		Model 3^c^	
	*F*	*P* value	*F*	*P* value	*F*	*P* value
CERAD-MS	4.62	.03	5.30	.02	3.04	.08
Frontal Assessment Battery	4.93	.03	5.60	.02	3.44	.06

Abbreviation: CERAD-MS, Consortium to Establish a Registry for Alzheimer Disease memory score, calculated by summing the participant’s word list recall test scores (maximum score, 10), the word list recognition test scores (maximum score, 10), and the average of 3 trials in the word list memory test (maximum score, 10).

^a^ Analysis of covariance adjusted for age and education.

^b^ Analysis of covariance adjusted for age, education, and factors that were not concordant within couples.

^c^ Analysis of covariance adjusted for age, education, factors that were not concordant within couples, and factors that were concordant within couples.

## Discussion

This study demonstrated that participants’ cognitive disorders were associated with spouses’ declines in cognitive function and risks of cognitive disorders, and that this association was mediated by factors including physical inactivity and a history of head injury, which were shared within couples. To our knowledge, this is the first study to reveal how the association among cognitive disorders, cognitive function, and shared risk factors is structured within couples.

The association of dementia with a spouse’s risk of dementia or cognitive decline has been studied extensively.^1,2,4,5,6,7,8,9,12,13^ A longitudinal study of older couples found that the spouses of persons who developed dementia had a 1.62 times greater risk of developing dementia than spouses of persons who did not develop dementia.^2,3^ Another study based on data from national registers reported that having a spouse affected by dementia increased the risk of incident Alzheimer dementia by 1.07 times.^1^ Both studies commonly indicated that a person’s cognitive disorder might be a risk factor for his or her spouse’s cognitive disorder. Cognitive disorder was also associated with spousal cognitive performance. Although impairments in multiple cognitive domains can lead to cognitive disorders, executive dysfunction and memory may be the leading causes of cognitive disorders associated within couples in response to several mediators such as depression and stress,^5,7,13^ which are in line with the current study.

The current study showed that the association between cognitive disorder and the risks of spousal cognitive disorders and cognitive impairments was mostly mediated by the factors shared within couples. Many previous studies proposed caregiving stresses as the main association factor because caregiving spouses are at a higher risk of cognitive impairment than noncaregiving spouses,^4,6,7,9^ and several factors have been suggested to mediate this association.^13^ According to the 2020 report of the Lancet Commission, 40% of dementia cases can be prevented or delayed by modification of 12 risk factors.^24^ Most of these factors, such as educational attainment,^42,43^ cardiometabolic disease,^17,19,20^ lifestyle factors,^17,18,21,44^ and depression,^15,45^ tend to be highly shared within couples with or without dementia. Furthermore, caring for a spouse with dementia may change the lifestyles shared within couples.^13^ In the current study, physical inactivity and MDD were significantly shared within couples, and physical inactivity mediated the shared risks of cognitive disorders and cognitive impairments through MDD. In many previous studies, physical inactivity was more likely to be shared within a couple,^18,21^ particularly in couples caring for spouses with dementia.^13^ Physical activity tends to decline from the prodromal stage of dementia,^46^ which may reduce a spouse’s physical activity. In the current study, physical inactivity was concordant within both the SCD+ and SCD− groups, but the concordance in the SCD+ group was stronger than that in the SCD− group. Physical inactivity and MDD are highly correlated. Low physical activity level is a potentially modifiable risk factor for depression, and physical activity can confer protection against the emergence of depression.^47^ In addition, individuals with depression may develop a more sedentary lifestyle and become less physically active.^48^ In our study, of these 2 highly correlated factors, only physical inactivity was associated with spousal cognitive disorder, and only MDD was associated with the risk of cognitive disorder. Although it is not possible to infer a causal relationship, our results suggest that spousal cognitive disorders could lead to a shared sedentary lifestyle within couples, which may be associated with the risk of cognitive disorders through association with depression. Therefore, encouraging physical activity in spouses of cognitively impaired patients may be a meaningful intervention to prevent cognitive disorders. Physical activity also has beneficial effects, such as stress modulation and its use as a coping resource for caregivers.^13^

In line with existing evidence,^24,49^ current MDD or a history of MDD was associated with cognitive disorders and cognitive performance in the present study. However, MDD did not mediate the association between cognitive disorders and a spouse’s risk of cognitive disorders or cognitive impairments in our study. Only 1 study has examined the mediating role of depression on the association between one’s cognitive disorder and a spouse’s cognitive performance.^7,11^ In that study, the caregiving spouses of patients with Alzheimer disease showed more subjective cognitive complaints, slower cognitive processing, and faster declines in cognitive processing speed during 2 years than the spouses of cognitively normal older adults, and the Hamilton Depression Rating Scale score mediated the differences between groups. However, the 2 groups showed comparable frequencies of depressive disorders diagnosed according to *DSM-IV* diagnostic criteria. Compared with structured clinical interviews based on diagnostic criteria, rating scales for depressive symptoms are overinclusive and have less specificity, particularly in older or physically ill populations.^50,51^

All grades of head injury, regardless of severity, are associated with increased risk of dementia, including Alzheimer disease.^24^ In addition, head injury was associated with a broad range of declines in cognitive functions, including memory and executive function.^52,53^ Head injury may promote the accumulation, misfolding, and aggregation of multiple abnormal proteins associated with neurodegeneration, leading to global reductions in brain volume via multiple mechanisms.^54^ However, none of the previous studies investigated spousal concordance of head injury or the associations between caregiving and the risk of head injury. In older people, falls are the leading cause of traumatic brain injury,^55,56^ and multiple factors, such as indoor home environment, physical frailty, compromised health status, and depressive symptoms, are associated with the risk of falls.^49,57,58^ These factors can be shared within couples and may increase the shared risk of head injury within couples. However, the incidence of head injury was low, the history of head injury was retrospectively collected with a questionnaire, and detailed information about head injury has not been sufficiently investigated. Therefore, the mediating role of head injury in shared cognitive disorders and impairments within couples needs to be confirmed by further studies on larger samples using detailed information on head injury.

### Limitations

This study had several limitations. First, it used a cross-sectional design. We cannot rule out reverse causality. Cognitive disorders and decreased cognitive functions shared within couples may influence the environments shared within couples, such as depression. That is, the mediating factors may be observed symptoms rather than risk factors. Second, this study could not analyze dementia and mild cognitive impairment separately because of the limited sample size. The mediating factors may be different between couples with mild cognitive impairment and those with dementia. Third, some of the potentially mediating factors, such as stress, anxiety, medication use, and caregiving status, were not considered. Fourth, we defined depression as a dichotomy rather than a continuous measure of depression, which might have affected the findings by the restriction of range. However, the responses to the Geriatric Depression Scale are known to vary between ethnic groups, which may considerably limit the generalizability of our results to other populations.

## Conclusion

This cohort study is, to our knowledge, the first to demonstrate that shared environments within couples may underlie shared cognitive disorders and cognitive performance within couples. Identification and intervention of the shared risk factors of dementia within couples may reduce the risk of cognitive disorders in the spouses of people with dementia.
